# Prognostic impact of absolute peripheral blood NK cell count after four cycles of R-CHOP-like regimen treatment in patients with diffuse large B cell lymphoma

**DOI:** 10.1007/s10238-023-01249-0

**Published:** 2023-11-08

**Authors:** Zhongjun Huo, Fang Chen, Jiajia Zhao, Ping Liu, Zhi Chao, Kang Liu, Ji Zhou, Dan Zhou, Lu Zhang, Haifeng Zhen, Wenqun Yang, Zhenqing Tan, Kaibo Zhu, Zimian Luo

**Affiliations:** 1 Department of Hematology, Central Hospital of Xiangtan, Xiangtan, 411100 China; 2 Department of Reproductive and Genetic Center, Central Hospital of Xiangtan, Xiangtan, 411100 China

**Keywords:** Diffuse large B cell lymphoma, Natural killer cell, R-CHOP chemotherapy, Survival

## Abstract

As a subtype of lymphocyte, natural killer (NK) cell is the first line of defense that shows a strong function in tumor immunotherapy response and clinical outcomes. The current study aims to investigate the prognostic influence of peripheral blood absolute NK cell count after four cycles of rituximab combined with cyclophosphamide, doxorubicin, vincristine and prednisone (R-CHOP) treatment (NKCC4) in diffuse large B cell lymphoma (DLBCL) patients. A total of 261 DLBCL patients treated with R-CHOP from January 2018 to September 2022 were enrolled. The low NKCC4 was observed in patients who died during the study period compared with survival individuals. A NKCC4 < 135 cells/μl had a remarkable negative influence in overall survival and progression-free survival (PFS) compared to a NKCC4 ≥ 135 cells/μl (*p* < 0.0001 and *p* < 0.0004, respectively). In addition, the OS and PFS were synergistically lower in a NKCC4 < 135 cells/μl group among DLBCL patients with GCB type or high IPI. In conclusion, this study indicates NCKK4 as a valuable marker in clinical practice and provides an insight for combination treatment of R-CHOP to improve outcomes of DLBCL patients.

## Introduction

Diffuse large B cell lymphoma (DLBCL) is the most prevalent subtype of non-Hodgkin’s lymphoma, accounting for approximately 35% worldwide, with an evaluated number of 27,650 new cases in the USA in 2016 [1–3]. Rituximab combined with cyclophosphamide, doxorubicin, vincristine and prednisone (R-CHOP) is considered as standard treatment for DLBCL populations regardless of the International Prognostic Index (IPI) [3]. Despite the prognosis of DLBCL patients improved significantly based on this modality, approximately 45–50% of cases occur relapse and the 10-year overall survival (OS) of elderly in the LNH03-6B trial is only 49.8% [3, 4], indicating a need for other meaningful prognostic markers for the guidance of clinical medication.

Lymphocyte count is found to be a significant prognostic parameter in DLBCL. A low absolute lymphocyte count in peripheral blood predicts poor survival in DLBCL treated with R-CHOP, particularly in the non-germinal center B cell-like (non-GCB) type [5–8]. As a subtype of lymphocyte, natural killer (NK) cell is the first line of defense that shows a strong function in tumor immunotherapy response and clinical outcomes [9]. Lee et al. have suggested NK cell count before R-CHOP therapy as a potential prognostic factor in de novo DLBCL patients [10]. Fingerle-Rowson et al. have found a low baseline absolute NK cell count in peripheral blood before R-CHOP treatment is associated with short-term progression-free survival (PFS) in DLBCL, but not OS [11]. However, the interference of absolute NK cell count in peripheral blood after R-CHOP treatment for prognosis in DLBCL patient is far from clear.

Herein, we investigate the association between absolute peripheral blood NK cell count after four cycles of R-CHOP (NKCC4) therapy and prognosis in DLBCL, indicating a low NKCC4 is related to poor PFS and OS in DLBCL, especially in GCB subtype.

## Patients and methods

### Patients

We retrospectively analyze all patients admitted to Xiangtan Central Hospital from January 2018 to September 2022, and they were histologically diagnosed with de novo DLBCL according to the World Health Organization classification. All enrolled patients received ≥ 4 cycles of R-CHOP or R-CHOP-like regimens treatment. Patients combined with additional hematological disease, malignancies, immune system disease, or with an expected survival of less than 3 months and therapy less than four cycles were excluded. The following baseline features were available including gender, age, Eastern Cooperative Oncology Group (ECOG) performance status, cell origin of DLBCL, Ann Arbor stage, International Prognostic Index (IPI) score, serum lactate dehydrogenase (LDH), bone marrow involvement and extranodal site. Absolute NKCC4 was calculated using flow cytometry. This study was approved by the Medical Ethics Committee of Xiangtan Central Hospital (Approval number: 2020-05-10), and all enrolled patients provided informed consent to receive treatment and written informed consent allowing the use of their medical records.

### Flow cytometry analysis

Absolute NKCC4 was centrally calculated from ethylene diamine tetraacetic acid (EDTA)-K2-anticoagulated peripheral blood using flow cytometry (FACSCanto II, BD). In brief, 2 ml of peripheral blood was obtained from DLBCL patients after four cycles of R-CHOP treatment and placed into EDTA-K2 located anticoagulant tube. 100 μl of whole blood was mixed with 10 μl of monoclonal antibody in the flow tube, followed by adding 500 μl of hemolysin. The count of NK cells, which were defined as CD3^−^CD56^+^CD16^+^ cells, was calculated using flow cytometry (normal range: 150–1100 cells/μl).

### Outcomes and follow-up

Treatment responses were determined according to the International Working Group Response Criteria for Malignant Lymphoma [12]. The primary endpoint was progression-free survival (PFS), measured from diagnosis to progression of disease, death, or the last follow-up. The secondary endpoints were OS, calculated from diagnosis to death or the last follow-up.

### Statistical analysis

Enumeration data were shown as frequency and percentage (%), and measurement data were represented as mean ± standard deviation (SD) and analyzed using Student’s *t* test. Pearson correlation analysis was performed to evaluate the correlation between NKCC4 and total lymphocyte count after four cycles of R-CHOP treatment. Optimal cutoff value of NKCC4 was detected as a biomarker of survival using receiver operating characteristic (ROC) curves. OS and PFS were analyzed using R.4.2.1, and statistical analysis was performed using the Kaplan–Meier method followed by the log-rank test. A *p* < 0.05 was considered to be statistically significant.

## Results

### Baseline characteristics of patients

A total of 261 DLBCL patients with median age of 64 (range, 27–88) were enrolled in this study. A total of 157 (60%) patients were older than 60 years, and the female/male ratio was 1.6:1. Additionally, 113 (43%) patients had an ECOG PS ≥ 2, 124 (48%) patients had elevated LDH, and 129 (49%) patients had an IPI score ≥ 3. A total of 168 (64%) patients had Ann Arbor stage ≥ III, and 183 (70%) patients belonged to non-GCB subtype. Extranodal site was involved in 160 (61%) patients, bulky disease was involved in 24 (9%) patients, and bone marrow involvement was involved in 33 (13%) patients. The baseline characteristics of all patients are shown in Table 1.

**Table 1:** Patient baseline characteristics

Characteristic	Total (n = 261)
Age	
Median (range)	64 (27–88)
Age > 60, *n* (%)	157 (60)
Sex, n (%)	
Female	161 (62)
Male	100 (38)
ECOG score	
0–1	148 (57)
2–3	113 (43)
Extranodal site, *n* (%)	
Yes	160 (61)
No	101 (39)
Bulky disease, *n* (%) (≥ 10 cm)	
Yes	24 (9)
No	237 (91)
Stage, *n* (%)	
I	36 (14)
II	57 (22)
III	75 (29)
IV	93 (34)
Elevated LDH, *n* (%)	
Yes	124 (48)
No	137 (42)
Bone marrow involvement	
Yes	33 (13)
No	228 (87)
IPI score	
0–2	132 (51)
3–5	129 (49)
Subtype *n* (%)	
GCB	78 (30)
Non-GCB	183 (70)
NKCC4, *n* (%)	
> 135 cell/μl	111 (43)
< 135 cell/μl	150 (57)

*ECOG* Eastern Cooperative Oncology Group; *LDH* serum lactate dehydrogenase; *IPI* International Prognostic Index; *GCB* germinal center B cell-like; *NKCC4* absolute peripheral blood NK cell count after four cycles of R-CHOP

### Therapeutic outcomes according NKCC4

**Fig. 1:**
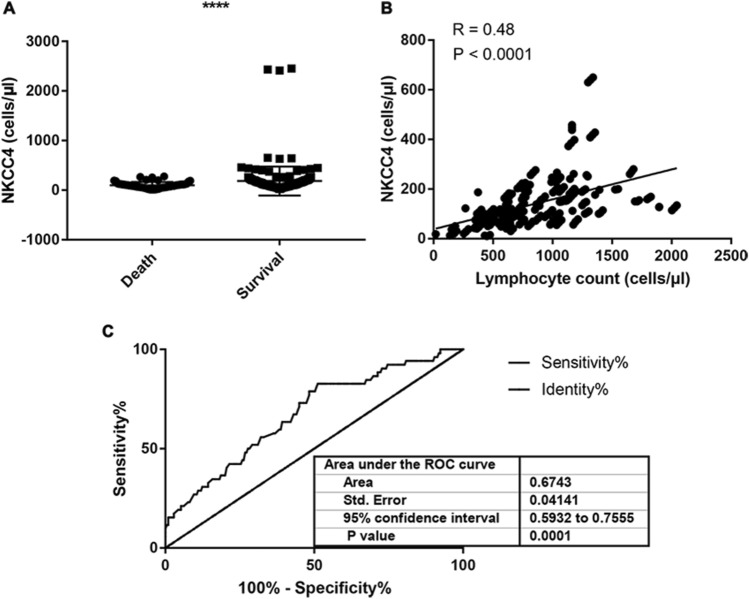
Box plot analysis and optimal cutoff value analysis for NKCC4 in this cohort. **A** Box plot analysis of the NKCC4 in DLBCL patients who were dead or remained survival until the last follow-up. The absolute NKCC4 was significantly higher in survival patients than those who died during the study period (*p* < 0.0001). **B** Pearson correlation analysis demonstrated the positive correlation between NKCC4 and total lymphocyte count after four cycles of R-CHOP treatment (exclude data with large discrepancies). **C** ROC curve analysis identified NKCC4 as a biomarker for death after R-CHOP therapy. Area under the ROC curve analysis suggested a cutoff value of 135 cells/μl (AUC = 0.67)

**Fig. 2:**
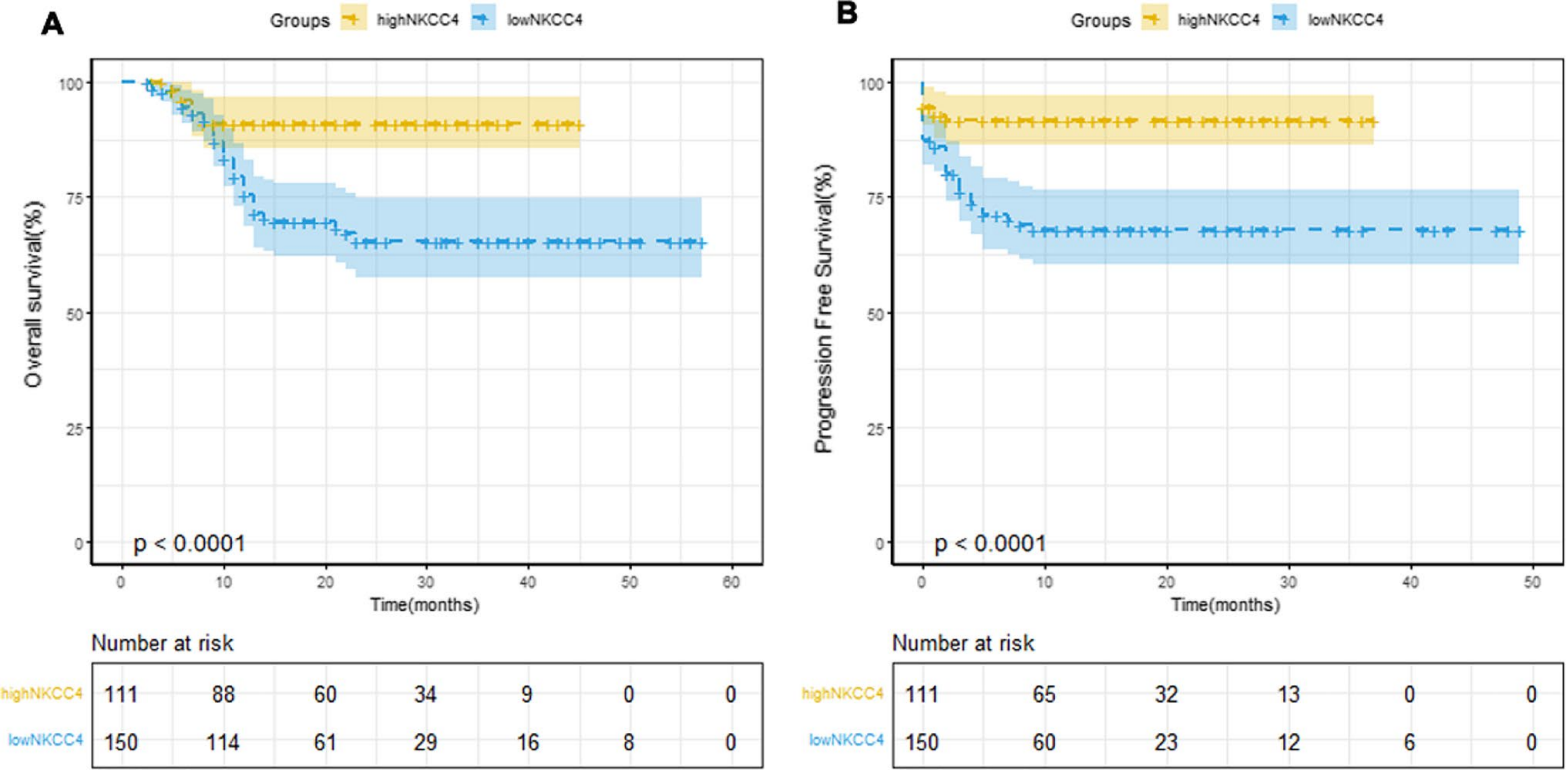
Therapeutic outcomes of patients according NKCC4. **A** OS and **B** PFS. Both OS and PFS rates of patients in the high NKCC4 group were significantly higher than those in the low NKCC4 group (*p* < 0.0001 for OS and *p* < 0.0004 for PFS)

All enrolled patients received ≥ 4 cycles of R-CHOP or R-CHOP-like regimens treatment, and absolute NKCC4 was examined. As shown in Fig. 1A, absolute NKCC4 was significantly higher in survival patients than those who died during the study period (*p* < 0.0001). In addition, Pearson correlation analysis demonstrated the positive correlation between NKCC4 and total lymphocyte count after four cycles of R-CHOP treatment (Fig. 1B). Furthermore, to evaluate the cutoff value of absolute NKCC4, area under the ROC curve (AUC) analysis were performed, demonstrating 135 cells/μl as a cutoff value (AUC = 0.67, *p* < 0.0001, Fig. 1C). This value was selected as an optimal cutoff value for subsequent analysis. A total of 150 (57%) patients showed high NKCC4 (< 135 cells/μl) (Table 1). Kaplan–Meier analyses demonstrated the significant high OS and PFS rates of patients in the high NKCC4 group compared with that in the low NKCC4 group (95% confidence interval (CI) = 0.15–0.46, *p* < 0.0001 and 95%CI = 0.073–0.33, *p* < 0.0004, respectively) (Fig. 2A and B).

### Survival according to the NKCC4 combined with prognostic factors

**Fig. 3:**
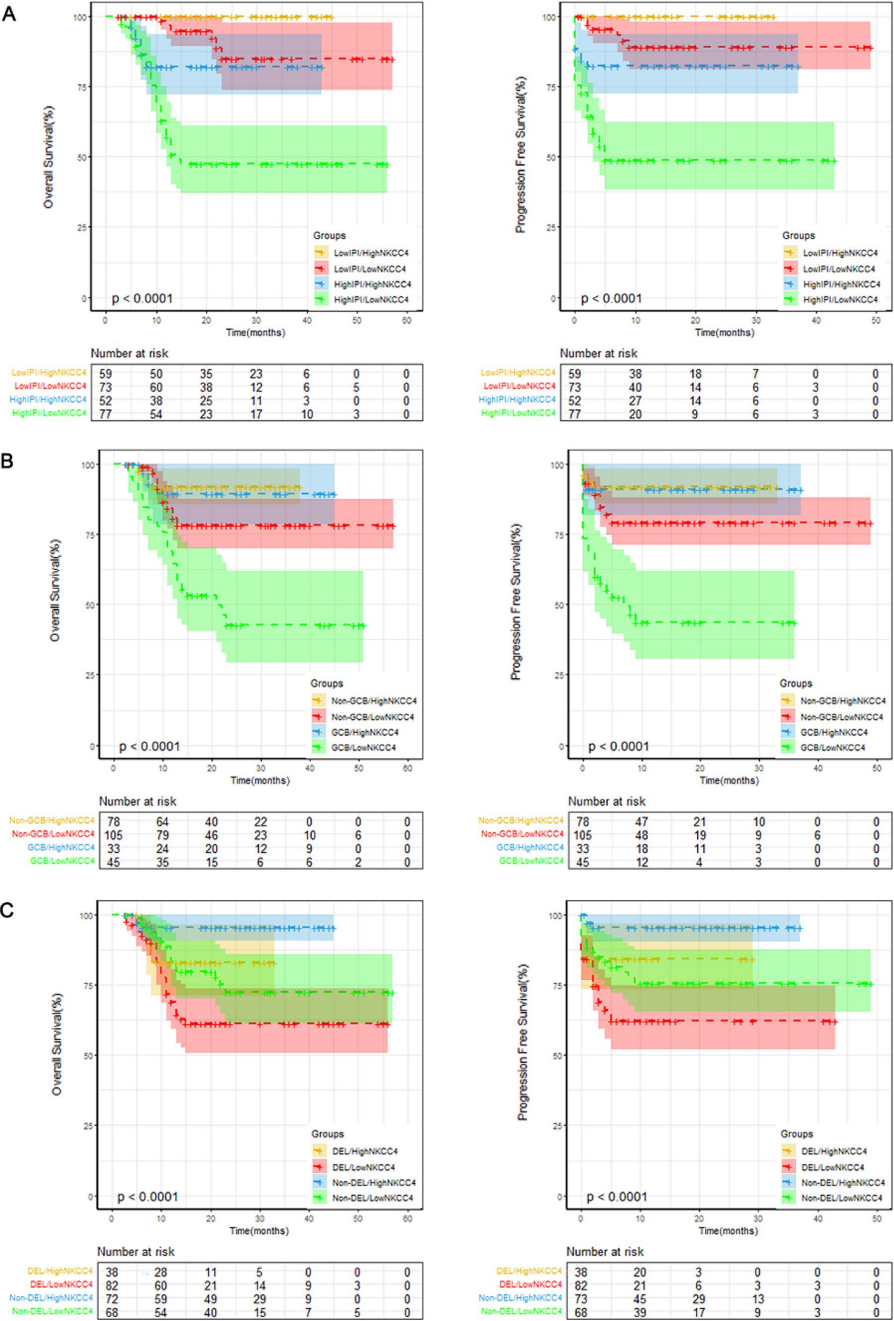
Survival according to the NKCC4 combined with prognostic factors. **A** OS and PFS according to the NKCC4 combined with IPI. **B** OS and PFS according to the NKCC4 combined with cell of origin. **C** OS and PFS according to the NKCC4 combined with DEL. The OS and PFS were synergistically lower in the low NKCC4 group among DLBCL patients with GCB type or high IPI

Furthermore, we explored the effect of NKCC4 in combination with cell for origin, IPI and co-expression of MYC and BCL2 (double-expressor lymphoma, DEL). Kaplan–Meier curves suggested that low NKCC4 obviously influences the OS and PFS rates in DLBCL patients with GCB or high IPI (Fig. 3). NKCC4 influenced survival outcomes in both patients with low and high IPI. Low NKCC4 was significantly decreased the OS or PFS rate, although the IPI was high (*p* < 0.0001) or low (*p* < 0.0001), compared to the high NKCC4 group (Fig. 3A). Both OS and PFS distinguished significantly refer to the NKCC4 among patients with GCB, but only OS among patients with Non-GCB. The OS and PFS of GCB DLBCL or OS of Non-GCB type were significantly lower in the low NKCC4 group compared to that in the high NKCC4 group (*p* = 0.0006 for OS of GCB type; *p* = 0.0011 for PFS of GCB type; *p* = 0.041 for OS of Non-GCB type). However, the PFS of Non-GCB type showed no statistical difference between low and high NKCC4 groups (*p* = 0.062) (Fig. 3B). In addition, low NKCC4 tended to affect PFS of DEL DLBCL (*p* = 0.0039) and OS of Non-DEL DLBCL (*p* = 0.0023), but not OS of DEL DLBCL (*p* = 0.068) and PFS of Non-DEL DLBCL (*p* = 0.0598), compared with the high NKCC4 group in our current cohort (Fig. 3C). Collectively, the present findings revealed that the OS and PFS were synergistically lower in the low NKCC4 group among DLBCL patients with GCB type or high IPI.

## Discussion

In the present study, we investigated the prognostic influence of peripheral blood absolute NK cell count in DLBCL patients treated with no less than four cycles of R-CHOP. Data analysis demonstrated the low expression of NKCC4 in patients who died during the study period compared with survival individuals, which was independently related to poor OS and PFS of DLBCL patients (cutoff value = 135 cells/μl). Previous study has demonstrated that antibody-dependent cellular cytotoxicity (ADCC) is a major mechanism of antitumor efficacy of clinically used antitumor antibodies including rituximab [13]. However, NK cells were considered as the primary effector of ADCC [14, 15]. Rituximab-mediated ADCC is well characterized and role for NK cell has been described [16]. As reported by Du et al., rituximab activates abundance of NK cells through overcoming the requirement for HLA-dependent NK cell licensing, thereby contributing to clinical responses in patients [17]. In addition, as effector cytotoxic lymphocytes, NK cells have been proposed as a diagnostic biomarker to predict prognosis of numerous malignancies including acute lymphoblastic leukemia [18], hepatocellular carcinoma [19] and gastric cancer [20], due to its immunomodulatory effects. Low baseline peripheral blood absolute NK cell count is associated with poor clinical outcomes of DLBCL and follicular lymphoma patients [11, 21, 22]. NK cell deficiency was found in high-risk myelodysplastic syndromes with, and its deficiency was significantly related to worse prognosis [23]. Low level or defectiveness of NK cells may lead to insufficient immunity against cancer cells, resulting in disease deterioration due to impaired immune surveillance [11, 23]. In addition, this work demonstrated the positive correlation between NKCC4 and total lymphocyte count after four cycles of R-CHOP treatment. As previous studies reported, an absolute lymphocyte count is found to be a strong prognostic factor in DLBCL and a low lymphocyte count in peripheral blood before and after R-CHOP therapy predicts short-term survival in DLBCL [6, 24, 25].

Furthermore, we found that low NKCC4 synergistically decreased the OS and PFS among DLBCL patients with high IPI. IPI is now still the most extensive utilized prognostic index in DLBCL, although in the era of rituximab, which notably improves patient cure ratio [26, 27]. However, the use of IPI, revised-IPI, or National Comprehensive Cancer Network IPI to classify high-risk subgroup with long-term survival less than 50% is limited [28]. Thus, finding more precise prognostic biomarkers is crucial to identify individuals at high risk for relapse or progression. Recently, a prediction model combining baseline metabolic tumor volume and IPI score obviously improved patient outcome prediction compared with presently utilized IPI score, especially extremely high-risk subgroup population [29, 30]. Kusano et al. delineated that a low absolute peripheral blood CD4^+^ T cell count at diagnosis in combination with high-risk improved disease progression synergistically in DLBCL [31]. Our current study provided a novel prognostic biomarker, low NKCC4 (< 135 cells/μl) that can be used to identify badly high-risk subset of DLCBL.

Moreover, we indicated the prognostic function of NKCC4 in GCB DLBCL, whereas no significant influence was found in PFS of Non-GCB subset, which is consistent with previous study, demonstrating the prognostic role of baseline NK cell count in GCB DLBCL [11]. The mechanism contributed to this distinction is unclear. It may be associated with the differences in their microenvironment and potential tumor genetics, given that special cell of origin subtypes exhibits different mutational patterns and is driven by distinct carcinogenic pathways [32, 33]. For instance, GCB subtype derives from lymphoid B cells in germinal center and usually expresses Bcl-6, CD10 and LMO2, with frequent genetic aberrations features of t(14;18) translocations (IGH-Bcl-2), *PTEN* deletion, *REL* amplification and mutations in epigenetic regulators, such as EZH2 [34–36], whereas Non-GCB DLBCL, which has a more unfavorable prognosis, is typically characterized by alterations in toll-like receptor and B cell receptor pathways in activated B cell subtype [37–39], and alterations in T cell response and JAK/STAT pathways are normally observed in primary mediastinal B cell lymphoma DLBCL [40].

In conclusion, our current findings demonstrated the low expression of NKCC4 in patients who died during the study period compared with survival individuals and identified NKCC4 with a cutoff value of 135 cells/μl as an independently prognostic marker for OS and PFS of DLBCL patients. In addition, we found that the OS and PFS were synergistically lower in the low NKCC4 group among DLBCL patients with GCB type or high IPI. The limitation of this study is the short follow-up. In addition, larger cohort should be conducted to verify our conclusion. Our study suggests NCKK4 as a valuable marker in clinical practice and provides an insight for combination treatment of R-CHOP to improve outcomes of DLBCL patients.

## Data Availability

The data that support the findings of this study are available on request from the corresponding author, [ZL] upon reasonable request.
